# AGING WITH SCHIZOPHRENIA: RACIAL DISPARITIES IN COGNITIVE IMPAIRMENT

**DOI:** 10.1093/geroni/igz038.1008

**Published:** 2019-11-08

**Authors:** Sarah Dobbins, Erin Hubbard, Heather C Leutwyler

**Affiliations:** 1 UCSF SChool of Nursing, San Francisco, California, United States; 2 UCSF, San Francisco, California, United States

## Abstract

Introduction: Cognitive impairment (CI) is a core feature of schizophrenia (SCZ). Comorbidities such as substances/smoking, metabolic syndrome, and medications contribute to CI. There are racial disparities in CI in the general US population. No study has evaluated CI racial disparities among people with schizophrenia (PWSCZ). The aims of this study were to describe the clinical/psychosocial correlates and racial disparities in CI. Methods: Cognitive performance in PWSCZ over 55 years old was measured using MATRICS Consensus Cognitive Battery (N=66). We calculated age- and gender-corrected scores for global cognitive performance, which represent number of standard deviations away from the mean of the MCCB normative sample. Clinical and sociodemographic data were collected. A counterfactual approach was used to explore mediation of CI through education. Results: Our “all-comer” convenience sample represented 57.6% white, 25.8% black, and 16.6% other non-white groups. There was a black/non-black disparity in cognitive score (-2.33 v.s. -1.68, t=2.843, p<.01). This difference remained significant in a regression model adjusted for age, substance use, smoking, education, antipsychotic medication, and positive/negative symptoms (-.6611, [95%CI:-1.12,-.20], overall F8, 57=3.690, p=.0016). In the mediation analysis, education accounted for 19% of the disparity in CI. In the counterfactual scenario in which education was distributed equally, education accounted for 48% of the disparity. Conclusion: There are significant racial disparities in cognitive performance among older PWSCZ, and educational attainment may account for a sizable portion of the disparity.

